# Supplementary material for: What does it take to learn the rules of RNA base pairing? A lot less than you may think
Source: bioRxiv. 2025 Aug 2:2025.07.31.668042. Preprint. [Version 1] doi: 10.1101/2025.07.31.668042 (PMC12324431; doi:10.1101/2025.07.31.668042)
Supplement: Supplement 1 [file media-1.gz › supplemental_material/src/rscape_v2.6.0.tar.gz!/rscape_v2.6.0/lib/infernal/Manuscripts/filter_thresholds/figs/4fam_score_hists.pdf]

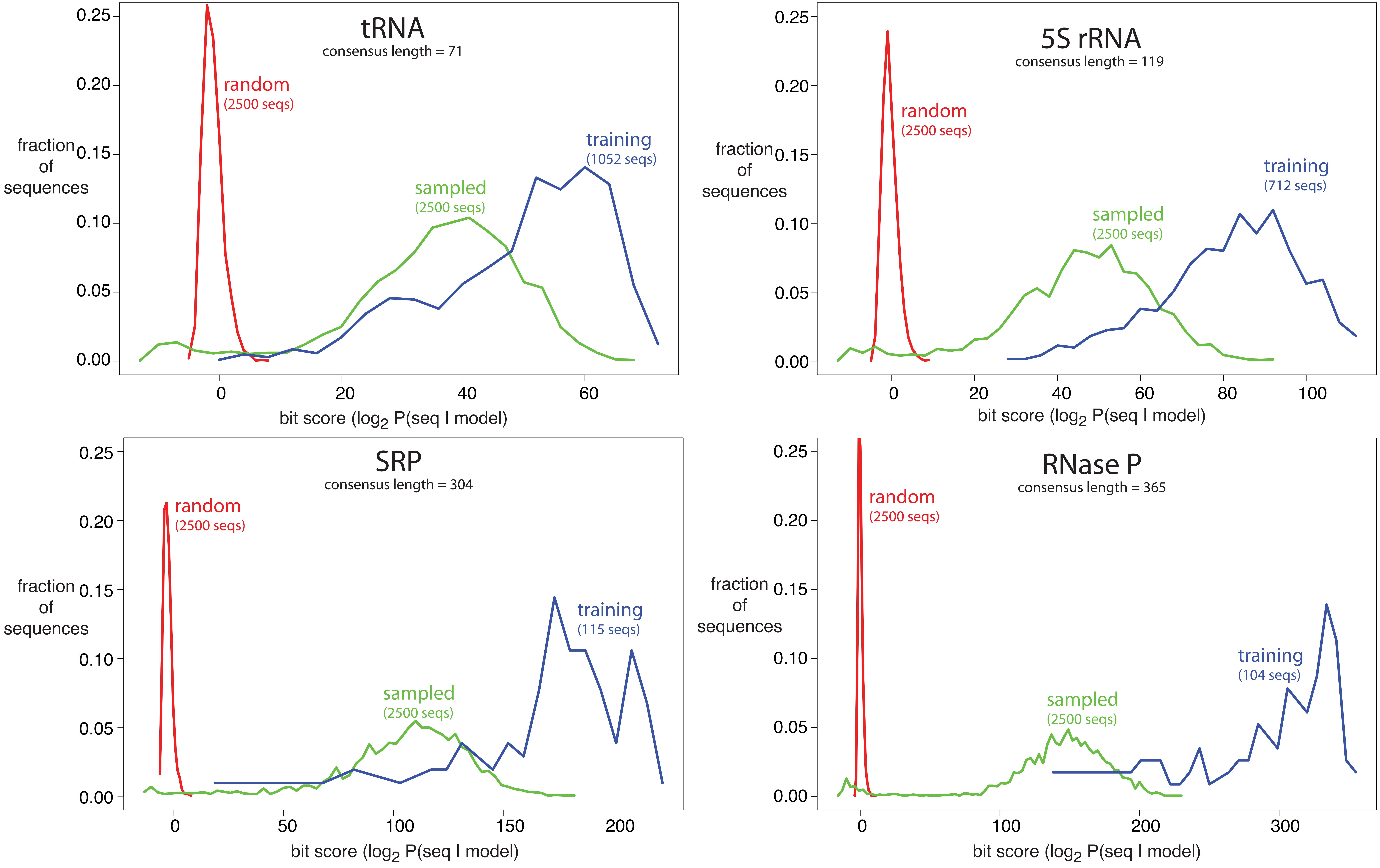

tRNA
consensus length = 71
random
(2500 seqs)
training
(1052 seqs)
sampled
(2500 seqs)
fraction of sequences
0.25
0.20
0.15
0.10
0.05
0.00
0
20
40
60
bit score (log2 P(seq I model)
5S rRNA
consensus length = 119
random
(2500 seqs)
training
(712 seqs)
sampled
(2500 seqs)
fraction of sequences
0.25
0.20
0.15
0.10
0.05
0.00
0
20
40
60
80
100
bit score (log2 P(seq I model)
SRP
consensus length = 304
random
(2500 seqs)
training
(115 seqs)
sampled
(2500 seqs)
fraction of sequences
0.25
0.20
0.15
0.10
0.05
0.00
0
50
100
150
200
bit score (log2 P(seq I model)
RNase P
consensus length = 365
random
(2500 seqs)
training
(104 seqs)
sampled
(2500 seqs)
fraction of sequences
0.25
0.20
0.15
0.10
0.05
0.00
0
100
200
300
bit score (log2 P(seq I model)
